# ﻿A new species of Zingiberaceae, *Curcuma glandulosa* (Subgenus Ecomatae), from Myanmar

**DOI:** 10.3897/phytokeys.266.163271

**Published:** 2025-11-25

**Authors:** Phyo Phyo Thwe, Mu Mu Aung, Nobuyuki Tanaka

**Affiliations:** 1 Forest Research Institute, Forest Department, Ministry of Natural Resources and Environmental Conservation, Yezin, Nay Pyi Taw, Myanmar Forest Research Institute, Forest Department, Ministry of Natural Resources and Environmental Conservation Yezin Myanmar; 2 Department of Botany, National Museum of Nature and Science, 4-1-1 Amakubo, Tsukuba, Ibaraki 305-0005, Japan National Museum of Nature and Science Tsukuba Japan

**Keywords:** Ginger, Myanmar, new taxon, Shan State, taxonomy

## Abstract

A new species, *Curcuma glandulosa* P.P. Thwe, M.M. Aung & Nob. Tanaka, is described from Taunggyi, southern Shan State, Myanmar. The new species is assignable to Curcuma subgenus Ecomatae, characterized by its epigynous glands, anther spurs, and lack of a conspicuous coma of sterile bracts. *Curcuma glandulosa* is distinguished by its red leafy stems, white bracts with red stripes, L-shaped anther spurs with densely short glandular hairs throughout, and a prominent anther crest. The leaves develop after blooming.

## ﻿Introduction

The genus *Curcuma* L. is the second largest genus in the Zingiberaceae and comprises more than 120 species that occur throughout tropical and subtropical Asia, from India to South China, Southeast Asia, Papua New Guinea, and northern Australia (Wu and Larsen 2000; Záveská et al. 2012; Leong-Škorničková and Saensouk 2023).

Three subgenera are currently accepted: subgenus Curcuma L., characterized by well-developed coma bracts and closed-type (bell-shaped) flowers with epigynous glands; subgen. Hitcheniopsis (Baker) K. Schum., characterized by the absence of epigynous glands and anther spurs; and subgen. Ecomatae Škorničk. & Šída f., with well-developed ligules and anther spurs and fertile bracts connate only at the base and without conspicuous coma bracts (Leong-Škorničková et al. 2015a).

Recently, several new species of Curcuma subgen. Ecomatae from Indochina and southern Yunnan, China, have been described, namely *C. newmanii* Škorničková, *C. xanthella* Škorničková, *C. corniculata* Škorničková, *C. flammea* Škorničková, *C. peramoena* Souvannakhoummane & Maknoi, *C. arida* Škorničková & N.S. Lyì, *C. sahuynhensis* Škorničková & N.S. Lyì, *C. lindstromii* Škorničk. & Soonthornk., *C. tongii* Y.H. Tan & L.X. Zhang, *C. woodii* N.H. Xia & J. Chen, *C. cotuana* Lưu, Škorničková & H.Đ. Trần, and *C. ignea* Ruchis. & Jenjitt. (Leong-Škorničková and Trần 2013; Leong-Škorničková et al. 2014, 2015b; Souvannakhoummane and Maknoi 2014; Chen et al. 2015; Lưu et al. 2017; Zhang et al. 2019). Thus far, at least 45 species of subgen. Ecomatae have been documented (Souvannakhoummane et al. 2025).

The subgen. Ecomatae is widely distributed from eastern Myanmar and southern China to Cambodia (Ruchisansakun and Jenjittikul 2023). The latest checklist of Myanmar seed plants (Kress et al. 2003) includes 27 species of *Curcuma*. Since then, *Curcuma kayahensis* Nob. Tanaka & M.M. Aung has been described from Myanmar. On the other hand, *C. stolonifera* Nob. Tanaka, K. Armstr. & M.M. Aung was originally described as a member of the subgen. Ecomatae (Tanaka et al. 2020), but it was misidentified based on its morphological characters and is currently placed in the subgen. Curcuma. Therefore, only two species of the subgen. Ecomatae—*C. candida* (Wall.) Techapr. & Škorničková and *C. kayahensis*—have been known from Myanmar so far.

Fieldwork in Shan State led us to collect an unidentified Curcuma assignable to the subgen. Ecomatae. After detailed investigation of its morphology, we describe it here as new to science.

## ﻿Materials and methods

Field surveys in Taunggyi, southern Shan State, Myanmar, were conducted in May–June 2023. Herbarium specimens, spirit collections (70% ethanol), and photographic data were collected in the field and examined. The protologues of all published names, along with other pertinent literature on *Curcuma*, were collated and reviewed. Type specimens were deposited at RAF and TNS. The description in the taxonomic treatment is based on both spirit materials and dried and pressed herbarium specimens. Measurements of reproductive organs were made from the spirit specimens kept in TNS. Terminology used in the description follows Beentje (2016). Herbarium acronyms followed Thiers (2016–continuously updated).

## ﻿Taxonomic treatment

### ﻿ Curcuma glandulosa

Taxon classificationPlantaeZingiberalesZingiberaceae

P.P.Thwe, M.M.Aung & Nob.Tanaka sp. nov.

6B11C90F-DDA0-597F-B72A-7F8759365EDF

urn:lsid:ipni.org:names:77372482-1

Fig. 1

#### Type.

**Myanmar** • Shan State: Taunggyi Bird Sanctuary, Taunggyi Township, Taunggyi District, 20°46'10"N, 97°03'22"E, 1660 m elev., 23 June 2023, *Nobuyuki Tanaka, Mu Mu Aung, Phyo Phyo Thwe MY6745* (holotype-TNS; isotype-RAF).

#### Diagnosis.

*Curcuma glandulosa* is similar to *C. sahuynhensis*; however, it is distinguished from it by a combination of the following characters: red leafy stems (vs. green), broadly lanceolate lamina developing after flowering (vs. ovate to elliptic and developing at the same time of flowering), white bracts with red stripes (vs. whitish to pale green at base with coral pink to red tinge), white anther (vs. yellow), and having prominent anther crest (vs. almost negligible).

#### Description.

Small rhizomatous herb, ca. 40 cm tall. ***Rhizome*** not thickened, bearing tubers; tubers ovoid, externally whitish brown, internally cream white. ***Leafy shoot*** emerged after or at the same time of blooming, leafy stem red; pseudostem with leaf sheath, red; sheathing bracts 1–4; petiole slender, ca. 25 cm long; leaf blade broadly lanceolate, ca. 20 cm long, ca 8 cm wide at early stage of development, base obtuse, entire along the margin, acute at the tip, glabrous on both surfaces, light green. ***Inflorescence*** lateral, arising directly from rhizomes before leaves develop, with 1–3 sheathing bracts; peduncle stout, 10–12 cm long, sheathing bracts reddish white, glabrous; spike 4–7 cm long, 3–5 cm diam; bracts 3–8 per inflorescence, 4.5–5.5 cm long, ca. 3 cm wide, broadly ovate, round or acute at the apex, white with numerous red stripes, puberulous towards the apex and on the edges; bracteoles indistinct, translucent white, glabrous, one per flower. ***Flower*** 5–5.5 cm long, exserted from bracts, with 2–3 cincinnus flowers; calyx tubular, ca. 2 cm long, ca. 1 cm wide, apex 3-toothed with unilateral incision, an incision 1–2 mm long, translucent, white, puberulous; floral tube white, expanded at apex, 3 cm long, puberulous; dorsal corolla lobe triangular-ovate, 2 cm long, 0.5 cm wide (the widest position), attenuate at the apex, slightly convex, white, slightly yellowish at apex, puberulous at apex edge, glabrous on both surfaces; lateral corolla lobes oblong, acute at apex, 1.5 cm long, 0.6 cm wide, glabrous, white; labellum 2–2.3 cm long, 1.7 cm wide, trullate to rhomboid, deeply incised at distal part, an incision ca. 1 cm, whitish yellow at base, lemon yellow at apex with bright yellow raised band running through the center, with two red lines along the band at base, sometimes red lines very weak, glabrous, margin puberulous; lateral staminodes ovate to rhomboid, 2 cm long, 0.9 cm wide, rounded at apex, glabrous, lemon yellow, lighter towards the base, base whitish; stamen 1.3–1.6 cm long; filament ca. 1 cm long, yellowish white, 0.3 cm wide at apex, 0.6 cm wide at base, with glandular hairs; anther curved, spurred, connective tissue white, glandular, spur L- shaped, anther spurs 3 mm long, parallel with acute apices, white; anther crest 1 mm long, curved backwards, deeply emarginate, yellowish white; thecae 5 mm long, dehiscing along entire length, pollen white; epigynous glands two, straight, ca. 5 mm long, ca. 0.5 mm in diameter, apex subulate, white, creamy-white at base; ovary sub-globose, 2–3 mm long, ca. 2 mm wide, trilocular, pubescent, white; style white, glabrous; stigma capitate, placed between anther spurs, ca. 0.8 mm long, 1 mm wide, light yellowish white; ostiole ciliate. ***Fruit and seeds*** not seen.

**Figure 1.**
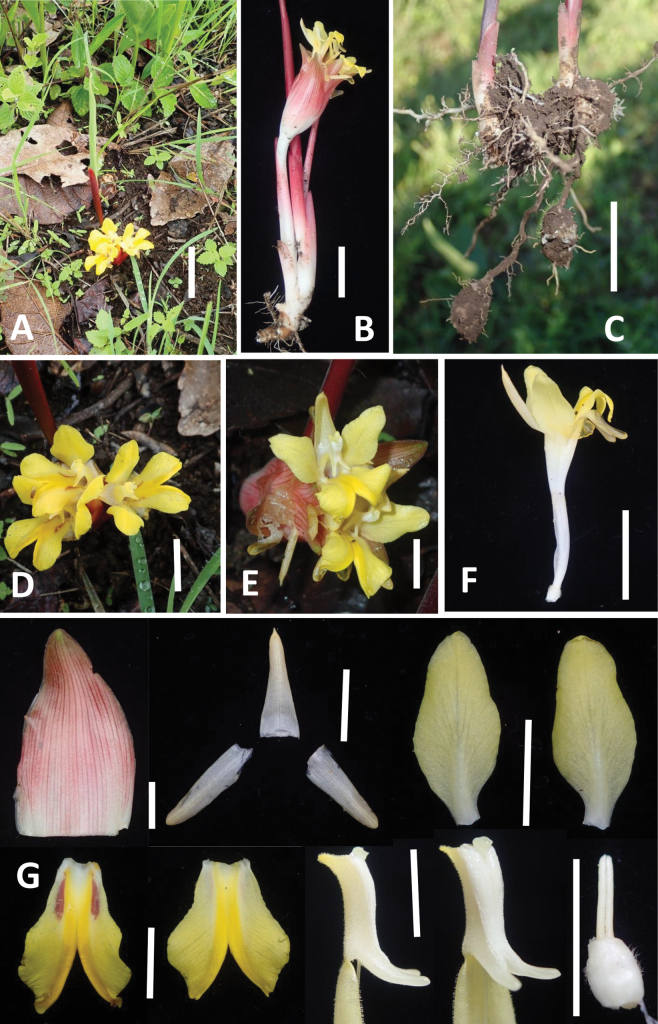
*Curcuma glandulosa* P.P. Thwe, M.M. Aung & Nob. Tanaka. **A.** Habit; **B.** Side view of inflorescence with flowers; **C.** Rhizomes with tubers; **D.** Flowers with prominent red lines on labellum; **E.** Flowers without or with weak red lines on labellum; **F.** Side view of a flower; **G.** Flower parts (from above left): bract, dorsal and lateral corolla lobes, lateral staminodes; (from lower left) labellum with red lines, labellum with very weak red lines, side view of stamen, diagonally front view of stamen, epigynous glands with ovary. Scale bars: 5 cm (**A**); 3 cm (**B**); 2 cm (**C–F**); 1 cm (**G**).

#### Etymology.

The specific epithet “*glandulosa*” refers to its character of densely glandular anther.

#### Distribution, habitat, and ecology.

Thus far known only from the type locality in Taunggyi, southern Shan State. However, it could be distributed in a wider range of limestone hills laying in eastern part of Myanmar. Growing in humid soil places at the edge of mix deciduous forests 1,500–1,600 m elevation. Inflorescence is arising before leafy shoot emerged.

#### Phenology.

Flowering in early rainy season from the end of May to mid-June. Fruits are not seen, and fruiting period is unknown. But we estimate that the fruit probably ripens during the rainy season from July to August.

#### Note.

*Curcuma glandulosa* also resembles *C. xanthella* Škorničk., described from Vietnam, but the new species is distinguished from it by having a sharply L-shaped anther (vs. gently L-shaped, crescent-shaped), a trullate to rhomboid labellum (vs. obovate), and shorter epigynous glands (5 mm vs. 15 mm).

## Supplementary Material

XML Treatment for Curcuma glandulosa

## ﻿Additional information

### Use of AI

No use of AI was reported.

### Author ORCIDs

Phyo Phyo Thwe https://orcid.org/0009-0009-5826-6614

Mu Mu Aung https://orcid.org/0000-0002-8263-9099

Nobuyuki Tanaka https://orcid.org/0000-0002-3003-7782

### Data availability

All of the data that support the findings of this study are available in the main text.
